# Academic anti CD19 CAR-T cell therapy for relapsed/refractory B-cell lymphomas in real-world clinical practice: a prospective cohort study

**DOI:** 10.3389/fonc.2026.1862517

**Published:** 2026-07-17

**Authors:** Natalya E. Konoplya, Tatiana M. Doroshenko, Katsiaryna Yu. Zharkova, Tatyana V. Savich, Ihar M. Seviaryn, Nadezhda M. Bobrova, Alexey A. Beleevsky, Eldar Kh. Sarydze, Sergey L. Polyakov

**Affiliations:** N. N. Alexandrov National Cancer Centre of Belarus, Minsk, Belarus

**Keywords:** academic CAR-T cell products, anti-CD19, cell therapy manufacturing, r/r B-cell lymphomas, real-world evidence, toxicity

## Abstract

**Background/objective:**

Chimeric antigen receptor (CAR) T-cell therapy has been the standard of care for relapsed/refractory B-cell lymphomas (r/r BCLs) in recent years. However, high cost of commercial products limits their application in real-world clinical practice. The use of the so-called academic CAR-T cell products can reduce the cost and improve availability and affordability of this therapy option. The aim of the present clinical trial was to assess the efficacy and safety of academic CAR-T cells in r/r BCL patients.

**Materials and methods:**

From June 2021 to December 2025, this prospective clinical trial enrolled 76 r/r BCL patients treated at the N.N. Alexandrov National Cancer Centre of Belarus (Minsk, Republic of Belarus), ClinicalTrials.gov Identifier: NCT07524816. The patients were 23–68 years of age (median 47 years). The CAR-T cell product was manufactured using lentiviral vector encoding anti-CD19 CAR.

**Results:**

The median follow-up was 7.85 months for the entire cohort and 20.12 months for patients without events. Generally, complete response was achieved in 57.5% of patients. The 4-year event-free survival (EFS) rate was 46.2 ± 6.3%, and the 4-year overall survival (OS) rate was 67 ± 5.9%. Among patients with follicular lymphoma (FL), complete response was achieved in 83.3% patients. The 4-year EFS was 81.7 ± 9.6%. The safety profile was acceptable: cytokine release syndrome (CRS) was detected in 43.4% of patients (≥ grade 3 in 2.6%). No cases of grade 4 CRS were reported, and neurotoxicity (ICANS) was identified in 26.3% of patients (≥ grade 3 in 5.3%, grade 4 ICANS occurred in two patients – 2.6%).

**Conclusion:**

The anti-CD19 CAR T-cell product demonstrates good efficacy and acceptable toxicity in patients with r/r BCL. The use of such a product is the only available option for patients in countries where commercial products are not accessible.

**Clinical trial registration:**

https://clinicaltrials.gov/study/NCT07524816, identifier NCT07524816.

## Introduction

CAR T-cell therapy has been the standard of care for r/r BCLs in recent years (1–3). It proved to be highly efficient, particularly in diffuse large B-cell lymphoma (DLBCL) and FL, providing complete response rates of 50–60% in patients with r/r DLBCL and leading to improved long-term survival (4–7). However, the high cost of commercially available products, such as axicabtagene ciloleucel, tisagenlecleucel, and lisocabtagene maraleucel, limits their widespread use in real-world clinical practice (8).

The diversity of clinical applications and the number of patients awaiting treatment are increasing exponentially. Consequently, the number of clinics and centers in need of the capacity to deliver CAR T-cell therapy is also growing.

Hence, several countries are exploring alternative T-cell production models that provide clinical outcomes comparable to commercial products (9). Access to CAR T-cells would be more equitable if they could be prepared in academic institutions, without the need for patient materials to be shipped and manufactured at centralized facilities. This model can be a realistic solution if it meets several criteria. The academic CAR T-cell platform should demonstrate successful and reproducible CAR T-cell manufacturing. Also the product should be comparable to approved cell therapeutics in terms of efficacy and toxicity. To be sustainable, this platform needs to involve a network of hospitals, scientists, and physicians willing to collaborate in the preparation and administration of academic CAR T-cells, with supervision of the network by a regulatory agency and the commitment to reimbursement of the academic CAR T-cell product. At the Hospital Clinic of Barcelona, a CD19 CAR T-cell product called ARI-001 for the treatment of patients with ALL and NHL and a BCMA CAR T-cell product called ARI-002h for patients with MM have been developed entirely through an academic network (10). Both products have been rigorously tested in clinical trials after receiving authorization from the Spanish Agency for Medicines and Health Products (AEMPS). Clinical results have shown efficacy and toxicity to be very similar to CAR T-cells prepared by industry or other academic institutions (11, 12). In this context, any other institutions worldwide may develop effective and safe academic CAR T cells within clinical trials, under a point-of-care (POC) national network. Some examples worldwide have been recently summarized, which highlights Canada and Brazil in addition to the EU (13).

Our study assesses the efficacy, safety, and long-term outcomes of investigator-initiated anti-CD19 CAR T-cell products in patients with r/r B- cell lymphomas, with particular emphasis on comparing our results with those from large clinical trials and real-world data. This comparison provides a rigorous evaluation of the academic CAR T-cells.

## Materials and methods

### Study design and participants

This prospective, non-randomized clinical study analyzed data from 76 patients treated between June 2021 and December 2025 at the N.N. Alexandrov National Cancer Centre of Belarus (Minsk, Republic of Belarus). The study was conducted in accordance with the Declaration of Helsinki and approved by the local ethics committee and included all patients meeting predefined eligibility criteria. The median patient age was 47 years (range: 23–68 years), with 50 (61%) male and 32 (39%) female patients.

Inclusion, exclusion and withdrawal criteria of the clinical trial (NCT07524816) are presented in **Supplementary Table**. In several cases, patients with ECOG 2–4 and patients with secondary CNS involvement were included as “compassionate use exceptions”. All participants provided written informed consent before enrollment in the study.

The most common histopathological subtype was DLBCL (n=42), including two cases with secondary central nervous system (CNS) involvement. Other subtypes included primary mediastinal large B-cell lymphoma (PMBCL; n=11), FL (n=18) and mantle cell lymphoma (MCL; n=5).

All patients underwent additional immunohistochemical analysis of tumor tissue to confirm CD19 expression prior to therapy initiation. High membranous CD19 expression (>50% of tumor cells) was observed in 80% of cases.

Patient characteristics for those with large B-cell lymphomas (DLBCL and PMBCL) are summarized in **Table 1**, while characteristics for patients with FL are presented in **Table 2**.

**Table 1:** Patient characteristics in r/r DLBCL and PMBCL (n =53).

Characteristic	Value, n (%) or median (range)
Male/Female	32 (60.4)/21 (39.6)
Age, years	47 (23–68)
ECOG performance status:	
< 2	37 (69.8)
≥ 2	16 (30.2)
DLBCL subtype:	
*De novo*	35 (83.3)
Transformed (from MCL, FL)	7 (16.7)
Cell of origin (Hans algorithm):	
GCB	5 (11.9)
Non-GCB	13 (31)
Unknown	24 (57.1)
MYC+	9 (21.4)
PMBCL	11(13.4)
Bulky disease (≥7.5 cm)	17 (32)
Disease refractoriness:	
Primary	49 (92.5)
Secondary	4 (7.5)
R-IPI score:	
0–2	24 (45.3)
3–5	29 (54.7)
prior HSCT:	
Allogeneic	1 (1.9)
Autologous	6 (11.3)
None	46 (86.8)
Prior therapy lines:	
<3	30 (56.6)
≥ 3	23 (43.4)
Bridging therapy	16 (30.2)
Elevated LDH	28 (52.8)
Elevated ferritin	30 (56.6)

ECOG, Eastern Cooperative Oncology Group; R-IPI, Revised International Prognostic Index; DLBCL, diffuse large B-cell lymphoma; LDH, lactate dehydrogenase; MCL, mantle cell lymphoma; PMBCL, primary mediastinal large B-cell lymphoma; HSCT, hematopoietic stem cell transplantation; FL, follicular lymphoma; GCB, germinal center B-cell-like.

**Table 2:** Patient Characteristics in FL (n = 18).

Characteristic	Value, n (%) or median (range)
Male/Female	10 (55.6)/8 (44.4)
Age, years	37 (30–67)
ECOG performance status:	
0–1	18 (100.0)
2	0
3–4	0
Disease refractoriness:	
Primary	17 (94.4)
Secondary	1 (5.6)
FLIPI score:	
0–1	4 (22.2)
2	9 (50)
3–5	5 (27.8)
HSCT:	
Allogeneic	0
Autologous	1 (5.6)
None	17 (94.4)
Prior therapy lines:	
≤2	10 (55.6)
>2	8 (44.4)
Bridging therapy	2 (11.1)
Elevated LDH	3 (16.7)
Elevated ferritin	6 (33.3)

ECOG, Eastern Cooperative Oncology Group; FLIPI, Follicular Lymphoma International Prognostic Index; LDH, lactate dehydrogenase; HSCT, hematopoietic stem cell transplantation.

Patient management consisted of three main components: manufacturing of the CAR T-cell product, conditioning (lymphodepletion), and CAR T-cell infusion.

### CAR-T

The academic CAR-T cell product presented in this study contains the anti-CD19 CAR construct with the single-chain variable fragment (scFv) of an anti-CD19 monoclonal antibody (FMC63) conjugated with the CD8 hinge region, 4-1BB transmembrane (TM), co-stimulatory domain, and the CD3ζ pro-activator signaling domain along with a truncated form of the epidermal growth factor receptor (EGFRt) cell surface protein as a co-expression marker and a safety switch mechanism. Monoclonal therapeutic antibody cetuximab binds EGFRt which effectively eliminates target cells (14). Product characterization, *in vivo* expansion, and persistence of CAR-T cells are presented in (15).

The required dose of CAR-T cells ranged from 0.5 × 10^6^ to 2.5 × 10^6^ cells/kg. However, in some patients, this target was not achieved. We made the decision to administer any available doses. Based on the administered dose, patients were stratified into the following groups: less than 1 × 10^5^ cells/kg, 1 × 10^5^–5 × 10^5^ cells/kg, and greater than 5 × 10^5^ cells/kg.

Persistence and expansion of anti-CD19 CAR-T cells *in vivo* was assessed by staining patients’ blood samples with anti-EGFRt cetuximab-like antibody conjugated with AlexaFluor 488 (RD Systems #FAB9577G). Samples were analyzed by flow cytometry every 3 days until day 30 and, subsequently, every 3 months. Cell product sterility was tested with BACT/ALERT microbial detection system and culture media bottles (bioMérieux) in a standard 7-day protocol. Viability of the CAR-T product was assessed by trypan blue exclusion. The CAR-T product was infused after passing sterility and viability (≥ 90%) tests.

Immunoglobulin G (IgG) concentration was measured using nephelometry (analyzer BN Prospec, Siemens) on days 0, 14, and 30, then every 3 months thereafter. B-cell aplasia was assessed in peripheral blood by flow cytometry (Beckman Coulter Navios), using a panel of antibodies including CD3, CD45, and CD19, on days 0, 14, and 30, and every 3 months thereafter. The duration of B-cell aplasia was calculated as the number of days post-CD19 CAR-T therapy until the B-cell population exceeded 1% of the total leukocyte population.

### Procedures

During the CAR T-cell manufacturing period, 21 of 76 patients (27.6%) received bridging therapy, including antibody-drug conjugates (brentuximab vedotin or polatuzumab vedotin) in 5 cases (23.8%), rituximab-containing chemotherapy regimens in 12 cases (57.1%), and radiotherapy in 4 cases (19%).

All patients received a lymphodepleting regimen consisting of intravenous fludarabine (30 mg/m² administered over 30 minutes on days -5, -4, and -3) and intravenous cyclophosphamide (300 mg/m² administered over 60 minutes on the same days). On day 0, anti-CD19 CAR T-cells were administered as a 30-minute intravenous infusion via a central venous catheter.

Primary endpoints included assessment of toxicity and overall response rate (ORR) evaluated by 2-deoxy-[18F]-fluoro-D-glucose positron emission tomography/computed tomography (FDG-PET/CT) performed on day 30 post-infusion. Secondary endpoints included EFS and OS. Subsequently, PET/CT scans were performed every 3 months.

The severity of CRS and ICANS was graded according to NCCN criteria (16). The first 10 patients received prophylactic tocilizumab before CAR-T infusion. Assessment of early hematological toxicity was conducted according to the ICAHT scale (17). Prior to the initiation of lymphodepletion, patients were stratified based on the risk of cytopenia and associated infections using the CAR-HEMATOTOX assessment system (18).

### Statistical analysis

EFS was defined as the time from CAR T-cell infusion to disease progression, relapse, or death from any cause, whichever occurred first; patients alive without events were censored at the last follow-up. OS was calculated from the date of infusion to the date of death or last follow-up. Survival probabilities were estimated using the Kaplan–Meier method, and survival curves were compared using the log-rank test. Multivariate Cox proportional hazards regression analysis was performed to identify independent prognostic factors significantly associated with EFS. The model included variables with potential clinical relevance based on univariate analysis and prior evidence. All statistical analyses were conducted using R software (version 4.3.1). A two-sided p-value < 0.05 was considered statistically significant.

## Results

**Figure 1:**
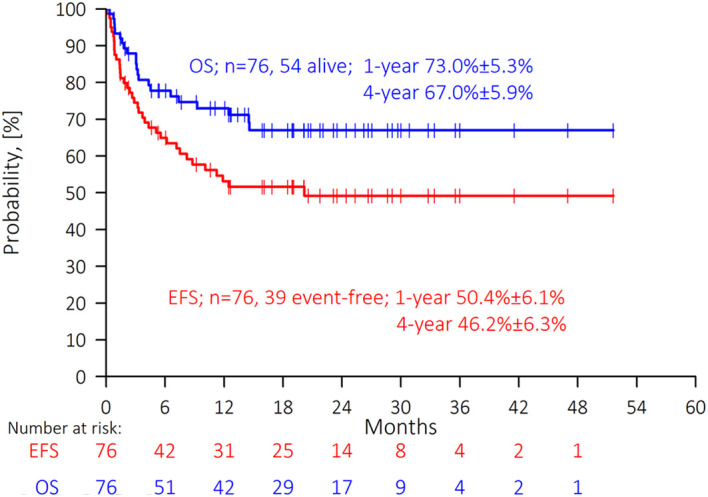
EFS and OS of r/r ВCL patients (*n* = 76).

In the overall cohort (n=76) a complete response (CR) was achieved in 57.5% patients, partial response (PR) in 21.9%, while stable disease and disease progression were observed in 2.7% and 17.8% of cases, respectively (3 patients died before the day-30 FDG-PET/CT assessment). The median follow-up was 7.85 months for the entire cohort and 20.12 months for patients without events. The 1-year EFS rate was 50.4% ± 6.1%, the 4-year EFS rate was 46.2 ± 6.3%. The 1-year OS rate was 73.0% ± 5.3%, the 4-year OS rate was 67 ± 5.9% (**Figure 1**).

**Figure 2:**
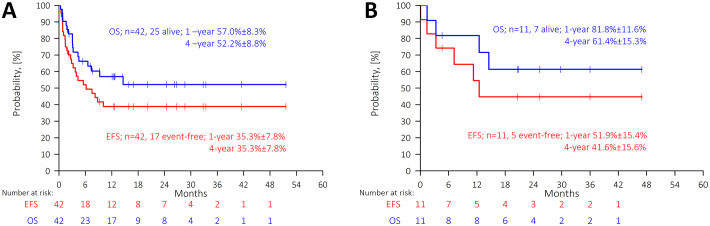
EFS and OS of patients with **(A)** r/r DLBCL (*n* = 42) and **(B)** PMBCL (*n* = 11).

In patients with DLBCL (n=42), CR was observed on day 30 in 51.3%, PR in 25.6%, stable disease in 5.1%, and disease progression in 17.9%. The median follow-up was 3.8 months for all patients and 16.9 months for patients without events. The 1-year and 4-year EFS rate was 35.3% ± 7.8%. The 1-year OS rate was 57.0% ± 8.3% and the 4-year OS rate was 52.2 ± 8.8% (**Figure 2A**).

In patients with PMBCL (n=11), CR was achieved in 36.4% of cases, PR in 36.4%, and disease progression in 27.3%. The median follow-up was 11.25 months for all patients in this subgroup and 25.35 months for patients without events. The 1-year EFS rate was 51.9% ± 15.4%, 4-year EFS rate was 41.6 ± 15.6%. The 1-year OS rate was 81.8% ± 11.6% and the 4-year OS rate was 61.4 ± 15.3% (**Figure 2B**).

**Figure 3:**
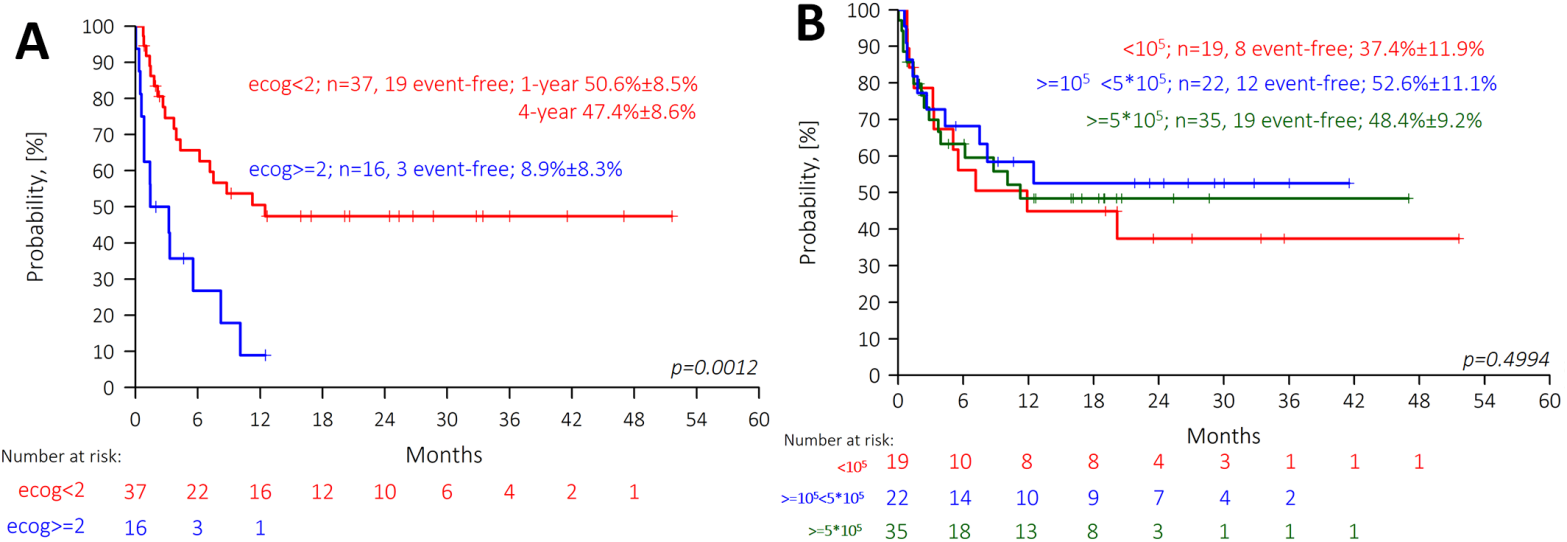
**(А)** EFS of patients with r/r DLBCL and PMBCL depending on their status: ECOG < 2 (*n* = 37) and ≥ 2 (*n* = 16), **(B)** EFS of patients with r/r BCL depending on their CAR-T dose.

Long-term outcomes were further analyzed in the combined subgroup of patients with DLBCL and PMBCL according to prognostic factors. Univariate analysis revealed that EFS was significantly associated with patient’s performance status (**Table 3**) (rates ​​are given for the median follow-up for the entire group of 7.8 months). Specifically, the 1-year EFS rate was 50.6% ± 8.5%, the 4-year EFS rate was 47.4 ± 8.6% in patients with an ECOG performance status of < 2, compared to a median EFS of only 1.5 months in those with ECOG ≥ 2 (p < 0.0012) (**Figure 3A**).

**Table 3:** Univariate analysis of prognostic factors (n=53).

Factors	n	EFS,% 7.85 months	P logrank	HR (95% CI)	p
Bulky					
Bulky -	36	48.1 ± 8.6	0.5822	1	0.5821
Bulky +	17	52.9 ± 12.1		1.2 (0.59 – 2.61)	
LDH					
LDH normal	25	56.0 ± 9.9	0.1077	1	0.1112
LDH elevated	28	45.5 ± 9.6		1.8 (0.87 – 3.76)	
ECOG					
ECOG <2	37	56.7 ± 8.5	0.0012	1	0.0020
ECOG >=2	16	26.8 ± 12.1		3.16 (1.52- 6.57)	
R- IPI					
R-IPI<3	23	52.2 ± 10.4	0.1067	1	0.0870
R-IPI >=3	29	46.1 ± 10.0		1.9 (0.91 – 4.00)	
Previous lines of therapy					
<3	30	36.7% ± 9.1	0.4747	1	0.4743
>=3	23	63.6% ± 10.3		0.77 (0.37-1.58)	

EFS, event free survival; HR, hazard ratio; LDH, lactate dehydrogenase; ECOG, Eastern Cooperative Oncology Group; R-IPI, Revised International Prognostic Index.

Furthermore, EFS was not associated with lactate dehydrogenase (LDH) levels: in patients with normal LDH (N ≤ 247U/l) (n = 25), the 4-year EFS rate was 56.0 ± 9.9%, compared to 45.5 ± 9.6% in those with LDH elevated 2–3 times above the upper limit of normal (n=28) (p = 0.112). Factors such as presence of bulky disease, number of prior lines of chemotherapy, and Revised International Prognostic Index (R-IPI) risk group did not have a significant impact on long-term survival outcomes in patients with r/r DLBCL and PMBCL (**Table 3**).

Since some of our patients previously received multiple lines of prior chemotherapy/targeted therapy, including bendamustine, which significantly suppresses the T-cell component (particularly the naive T-cells subset) and is the primary cause of manufacturing failure (19), it was not always possible to obtain a substantial dose of the cellular product ≥0.5×10^6^/kg. We link it to the fact that 26.3% (n=20) of our patients had ≤5% naive T-cells in CD3-positive cell population. However, according to our study protocol, patients were infused with any dose of CAR-T cells that met the quality control criteria for sterility and viability.

To analyze the influence of the CAR-T cell dose received by patients on therapy efficacy outcomes, we divided the entire patient cohort (n=76) into three groups based on the administered dose: less than 1×10^5^ cells/kg (n=19), 1×10^5^–5×10^5^ cells/kg (n=22), and greater than 5×10^5^ cells/kg (n=35) (**Figure 3B**). Statistical analysis showed no significant difference between these groups (p=0.4994), indicating that CAR-T cells achieve an effective therapeutic dose as a cellular product via robust expansion upon encountering their target in the patient’s body.

However, the analysis of adverse events related to CAR-T therapy showed that the incidence of ICANS grade I and II differed statistically (p=0.012) between the groups receiving less than 5×10^5^ cells/kg of CAR-T (9.8%) and more than 5×10^5^ cells/kg (34.3%) (data not shown). It is important to note that no significant differences were found in the frequency of severe grade III and IV ICANS (9.8% versus 5.7%, respectively; p=0.68) (data not shown).

**Figure 4:**
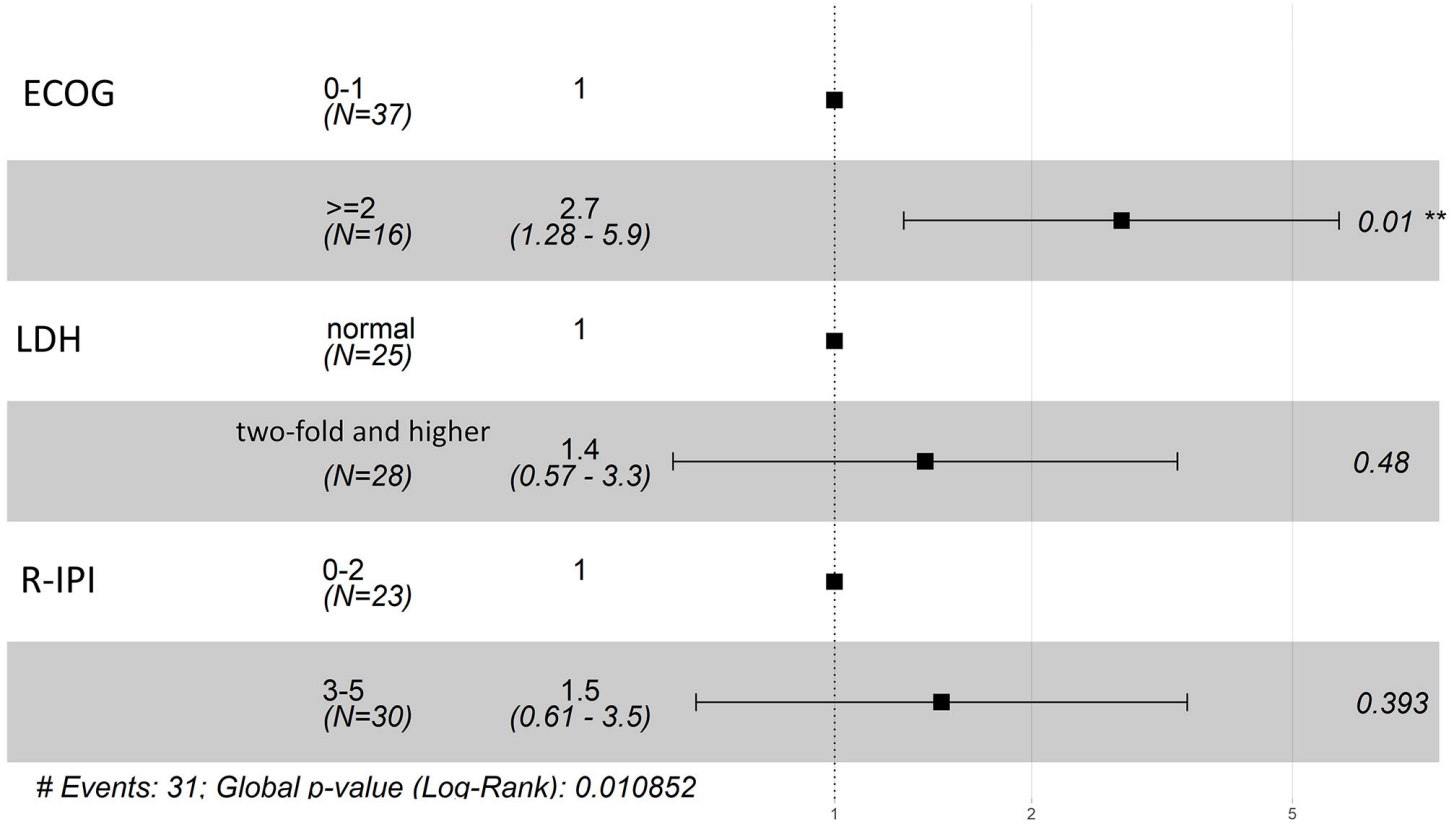
The results of multivariate Cox regression analysis for EFS. 95% confidence interval; ECOG, Eastern Cooperative Oncology Group; LDH, lactate dehydrogenase; HR, hazard ratio; R-IPI, revised international prognostic index.

Multivariate Cox regression analysis identified ECOG performance status as independent unfavorable prognostic factor significantly associated with the rate of EFS (**Figure 4**).

**Figure 5:**
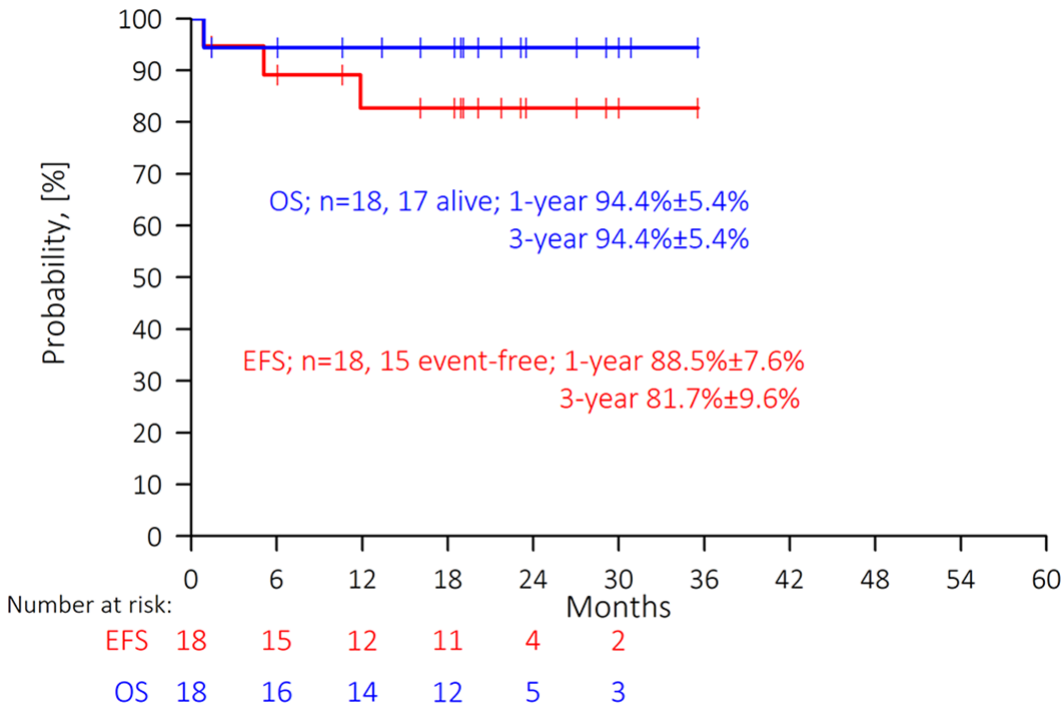
EFS and OS of patients with FL.

In patients with FL (n=18), CR was achieved in 83.3% of cases, PR in 5.6%, and disease progression occurred in 11.1%. The 1-year OS rate, as well as the 3-year OS rate, was 94.4 ± 5.4%. The 1-year EFS rate was 88.5% ± 7.6% and the 3-year EFS rate was 81.7 ± 9.6% (**Figure 5**).

In our study, non-relapse mortality (NRM), defined as death unrelated to disease recurrence or progression, was 7.9% (6 out of 76 patients) (**Table 4**). The main causes of death were sepsis (n=2), acute heart failure (n=1), and acute pulmonary failure (n=1). In two patients the cause of death remained undetermined.

**Table 4:** Results of treatment (n=76).

Outcomes	n	%
Total	76	100.0
Progression	15	19.7
Relapse	16	21.1
NRM	6	7.9
Еvent-free	39	51.3

NRM, non-relapse mortality.

In patients with MCL (n=5), 2 patients had progression, 1 relapsed, and 2 patients are alive without an event.

CRS was observed in 43.4% of 76 patients, ≥ grade 3 in 2.6%. No cases of grade 4 CRS were reported. ICANS developed in 31.6% patients, ≥grade 3 in 5.3%. Grade 4 ICANS occurred in two patients.

Concomitant ICANS and CRS were observed in 16 (21%) patients. Among them, two patients experienced infectious complications: one developed pulmonary embolism and one developed acute kidney injury.

In the assessment of early hematological toxicity using the ICAHT scale, which evaluates both the severity and duration of neutropenia, grade 1 (mild) neutropenia was observed in 36.1% ofxpatients, with neutropenia lasting less than 7 days; grade 2 (moderate) neutropenia occurred in 38.3% of patients, lasting 7–14 days; grade 3 (severe) neutropenia was observed in 21.3%, with duration exceeding 14 days. Additionally, two patients (4.2%) experienced grade 4 hematological toxicity, characterized by profound and prolonged neutropenia.

**Figure 6:**
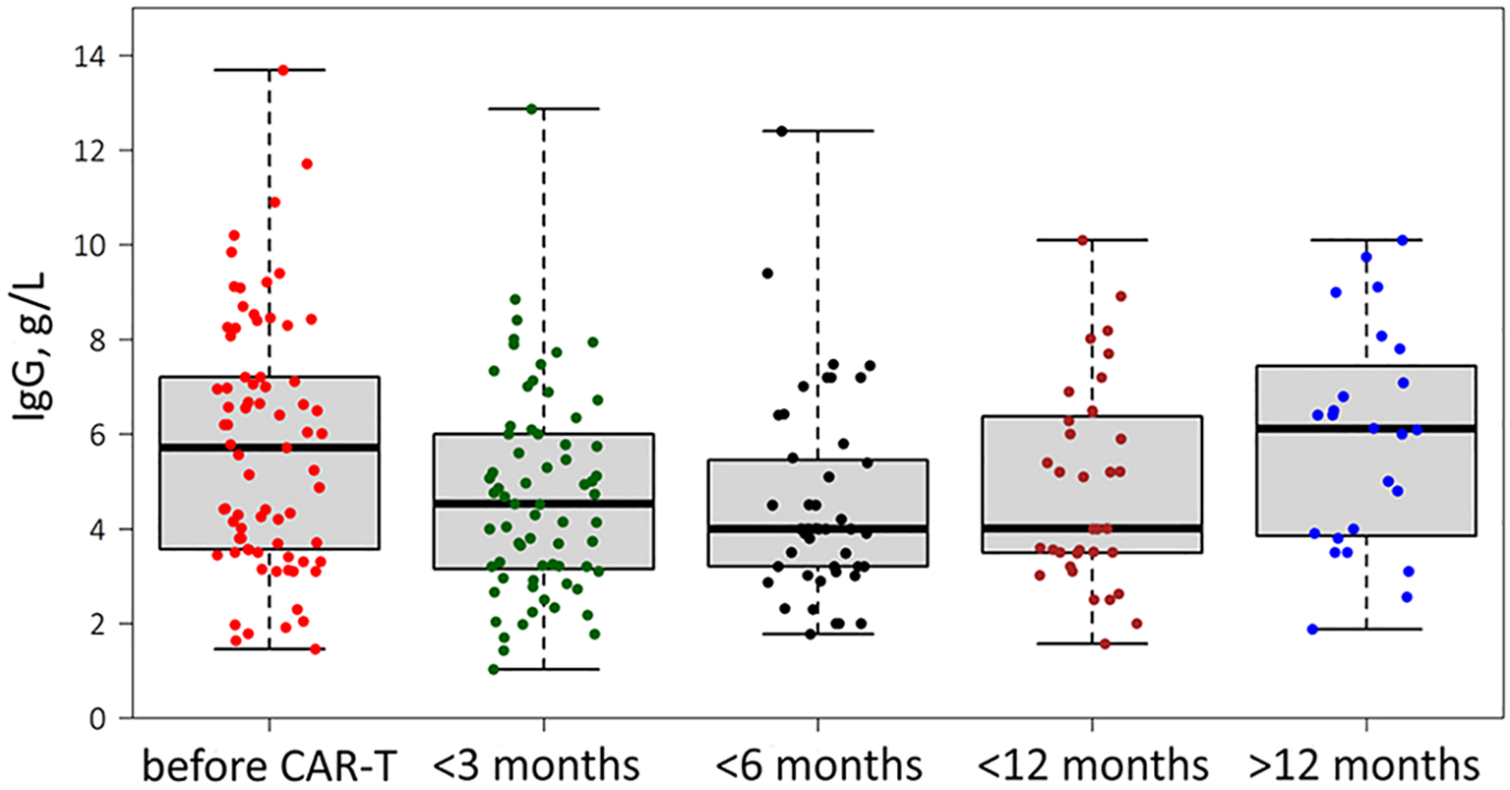
IgG levels before and after CAR-T therapy.

IgG levels were determined before the start of CAR-T cell therapy. 51.4% of patients had hypogammaglobulinemia (IgG ≤6 g/L) before treatment. A decrease in IgG levels was observed in 79.9% of patients within the first three months after therapy, remaining unchanged in a similar proportion of patients at six months (78%). At 12 months or more after CAR-T cell administration, hypogammaglobulinemia persisted in 49.9% of patients. Median IgG levels significantly decreased after CAR-T therapy (before and after: 5.67 g/L versus 4.8 g/L; p = 0.001) and persisted for 6 months after CAR-T therapy (p = 0.011) (**Figure 6**).

The median duration of B-cell aplasia was 300 days (interquartile range: 90–539). Infectious complications developed in 22.9% of patients. Upper respiratory tract infections were the most common type, accounting for 76.5% of all cases. All these patients received immunoglobulin replacement therapy during the observation period. The average IgG level before replacement therapy was 2.9 g/L and raised to 8.5 g/L upon treatment. The usual number of immunoglobulin infusions was three per patient.

## Discussion

The results of our study demonstrate the efficacy of academic CAR T-cell therapy in patients with r/r BCL, consistent with data from multicenter clinical trials. A CR was achieved in 51.3% of patients with DLBCL, which is comparable to outcomes reported in pivotal international studies.CR was observed in 58% of patients in the ZUMA-1 trial (axicabtagene ciloleucel) and in 53% of patients in the TRANSCEND trial (lisocabtagene maraleucel). The JULIET trial (tisagenlecleucel) reported lower response rates (40%) (3, 6, 20). According to real-world data by E. Bachy et al., CR rates typically range between 50% and 60% (21).

The 4-year OS in our study was 67.0 ± 5.9%, comparable with the 47% OS reported in ZUMA-1 and the 53% observed in TRANSCEND (3, 20). In real-world clinical practice, long-term survival rates generally range from 45% to 55%. It is important to note that patients in our cohort had less favorable baseline characteristics, including a higher proportion with ECOG performance status ≥2, which may have negatively influenced long-term outcomes.

A key objective of our study was to evaluate various adverse prognostic factors potentially affecting long-term survival. The analysis revealed both similarities and notable differences with large international CAR T-cell therapy trials. In our cohort, only poor ECOG performance status was a significant predictor of unfavorable outcomes. Patients with good performance status (ECOG 0–1) showed markedly better survival: the 4-year EFS was 47.4 ± 8.6% in this group, whereas median EFS was only 1.5 months for patients with ECOG 2–4. These findings are fully consistent with the ZUMA-1 trial, in which 2-year progression-free survival was 60% for ECOG 0–1 versus 30% for ECOG ≥2 (20).

It should be noted that some well-established prognostic factors did not reach statistical significance in our analysis. Tumor burden, which in ZUMA-5 was associated with a 2.3-fold increased risk of death (p = 0.03) (22), showed only a trend toward worse outcomes in our cohort (p = 0.6).

Multivariate Cox regression analysis identified only the ECOG performance status as independent unfavorable prognostic factor significantly associated with the rate of EFS.

Our study of academic CAR T-cell therapy in r/r FL demonstrated excellent outcomes matching those of key clinical trials. CR was achieved in 83.3% of patients, which is identical to results from the ZUMA-5 trial with axicabtagene ciloleucel (80%) and comparable with ELARA trial with tisagenlecleucel (69%) (22, 23). In real-world studies, CR rates typically range from 60% to 75%, which is slightly lower than our findings. The number of prior therapy lines – a key predictor in ELARA (40% reduction in OS for patients receiving >3 lines) – did not reach statistical significance in our cohort, despite 41% of patients having received more than two prior regimens. The 3-year OS in our study was 94.4 ± 5.4%, which corresponds to others studies such as ELARA (82%) and ZUMA-5 (85%) (22, 23). In real-world practice, this metric typically ranges from 75% to 85%. Patient characteristics analysis revealed several favorable features in our cohort that may have contributed to superior outcomes: all patients had good performance status (ECOG 0–1), only 20% had elevated LDH (versus 30–40% in other trials), and fewer patients had received more than two prior therapies (46.7% vs. 60–70% in ELARA and ZUMA-5). While these factors undoubtedly influenced outcomes, they do not completely explain the magnitude of improvement observed.

Another critical aspect of our study was the safety profile of CAR T-cell therapy. We observed a favorable toxicity profile for academic CAR T-cells. CRS was observed in 43.4% of 76 patients, ≥ grade 3 in 2.6%. No cases of grade 4 CRS were reported. ICANS developed in 31.6% patients, ≥ grade 3 in 5.3%. Grade 4 ICANS occurred in two patients. Concomitant ICANS and CRS were observed in 16 (21%) patients.

Notably, the toxicity rates in our study were closer to those observed in major trials: ≥ grade 3 CRS and ICANS were reported in 13% and 31% of ZUMA-1 patients, respectively. Higher than grade 3 CRS was observed in 2% of patients and ≥ grade 3 ICANS in 4% of patients in TRANSCEND (3, 20). Real-world data suggest average rates of ≥grade 3 CRS of 10–15% and ≥ grade 3 ICANS of 15–25% (21).

We also analyzed the potential impact of the administered CAR-T cell dose on clinical outcomes of patients. Our findings align with data obtained from the use of commercial “out-of-specification” anti-CD19 CAR-T products in pediatric populations with B-cell acute lymphoblastic leukemia (B-ALL) and B-NHL (24), Japanese adult patients with B-cell ALL and lymphoma (25), as well as UK populations with LBCL (19), which demonstrate comparable clinical efficacy to in-specification CAR-T cells.

Regarding early hematological toxicity, 12 patients (25.5%) experienced severe toxicity (grades 3-4) according to the ICAHT scale. Of these, 7 patients (63.6%) had a CAR-HEMATOTOX score ≥2 (high risk). This indicates that nearly two-thirds of patients with the most severe hematotoxicity were preemptively identified as high risk by the CAR-HEMATOTOX scale, which is associated with an increased incidence of severe and prolonged neutropenia.

Within the first 30 days post-CAR T-cell infusion, three serious infectious events were recorded. Two patients (2.8% of the total cohort), with grade 4 neutropenia, developed sepsis caused by *Acinetobacter baumannii*, resulting in death. The third patient was diagnosed with bilateral pneumonia on day 8 after infusion, requiring modification of antibacterial therapy with reserve antibiotics. All three patients were pre-classified as high risk (score ≥2) according to the CAR-HEMATOTOX scale, underscoring its prognostic value not only for hematotoxicity but also for associated life-threatening infections. No long-term hematological toxicity was observed.

Hypogammaglobulinemia is the most common on-target, off-tumor effect associated with CD19 CAR-T therapy. We found that hypogammaglobulinemia occurred in 78% of patients who received CAR−T therapy within six months, and in 72.9% within one year. It remained for more than 12 months after treatment. Infectious complications developed in 22.9% of patients. A common approach is immunoglobulin replacement for hypogammaglobulinemia (4 g/L) in severe or recurrent/chronic infections.

High cost and limited accessibility remain major challenges for CAR T-cell therapy. Our study made use of academic CAR T cells, which have the potential to reduce treatment costs. As demonstrated by C. Parker et al., academic CAR T-cells can be more cost-effective compared with commercial products such as tisagenlecleucel and axicabtagene ciloleucel (8). The potential of academic CAR T-cells also lies in their adaptability to local clinical needs and patient-specific requirements.

However, despite these advantages, academic CAR T-cell programs face challenges. Manufacturing requires strict quality control and adherence to Good Manufacturing Practice (GMP) standards (26). Moreover, long-term efficacy and safety data from academic products require further validation in larger prospective trials. Academic CAR T-cell therapy is a promising strategy to reduce costs and improve accessibility particularly in resource-limited settings. Also, in countries, where commercial products are not accessible, the use of such a product is the only available option for patients (27). Nevertheless, further research is needed to optimize efficacy, safety, and scalability of this approach. Limitations of our trial include the small number of patients, heterogeneous lymphoma subtypes, and differences in the prior chemotherapy treatment protocols. The results of this study confirm the efficacy and favorable safety profile of CAR T-cell therapy in patients with r/r BCL.

The use of academically manufactured CAR T-cell products represents a highly promising approach that may reduce the cost of treatment and improve accessibility, which is particularly critical for healthcare systems with limited resources (27). However, further research is needed to optimize the efficacy, safety, and scalability of this therapeutic modality to fully realize its potential in routine clinical practice.

## Data Availability

The raw data supporting the conclusions of this article will be made available by the authors, without undue reservation.
