# Supplementary material for: Academic anti CD19 CAR-T cell therapy for relapsed/refractory B-cell lymphomas in real-world clinical practice: a prospective cohort study
Source: Front Oncol. 2026 Jul 17;16:1862517. doi: 10.3389/fonc.2026.1862517 (PMC13423673; doi:10.3389/fonc.2026.1862517)
Supplement: Supplementary file 1 [file Table1.docx]

**Supplementary Table: Inclusion, exclusion and withdrawal and criteria of the Clinical trial**

| **Inclusion criteria** | 1. Presence of a multidisciplinary consensus indicating the absence of alternative curative treatment options and the necessity for cell therapy-based treatment as the only viable modality. 2. Written informed consent from the patient to participate in the study. 3. Patient compliance with the study protocol. 4. Patients aged 18 years or older. 5. Histologically confirmed aggressive B-cell non-Hodgkin lymphoma, including the following types as defined by the WHO 2016 classification: unspecified DLBCL; T-cell/histiocyte-rich large B-cell lymphoma; associated with chronic inflammation; EBV-positive DLBCL, unspecified; primary cutaneous DLBCL of the leg type; high-grade B-cell lymphoma with MYC and BCL2 and/or BCL6 rearrangements (double/triple-hit); primary mediastinal large B-cell lymphoma (thymic type); transformation of follicular lymphoma to DLBCL. 6. Relapsed or refractory disease after ≥2 lines of chemotherapy, including rituximab and anthracyclines, or relapse post-autologous hematopoietic stem cell transplantation (auto-HSCT). 7. Measurable disease at the time of inclusion:    1. Lymph node lesions >20 mm in the longest diameter, regardless of short axis length.    2. Extranodal lesions (outside lymph nodes but including liver and spleen) ≥10 mm in the longest and shortest axes (response assessment criteria for diffuse large B-cell lymphoma and follicular lymphoma). 8. Estimated life expectancy of ≥12 weeks. 9. Performance status according to the Eastern Cooperative Oncology Group (ECOG) scale of 0–1. 10. Expression of CD19 antigen on tumor cells (archived histological blocks, biopsy required if archival tissue is unavailable). 11. Adequate organ function based on the results of the following assessments:     1. Renal function:   Serum creatinine ≤1.5 × ULN or calculated creatinine clearance (CrCl) ≥45 mL/min (using the Cockcroft-Gault formula).   - 1. Hepatic function:   Alkaline phosphatase, alanine aminotransferase (ALT), and aspartate aminotransferase (AST) levels ≤5 × ULN; Total bilirubin ≤1.5 × ULN, except in cases of Gilbert's syndrome with transient elevation of unconjugated bilirubin.   - 1. Pulmonary reserve:   11.4 .Dyspnea of ≤Grade 1 and pulse oximetry >91% oxygen saturation at room air.  11.5 .Hemodynamic stability and left ventricular ejection fraction (LVEF) ≥45%, confirmed by echocardiogram or MUGA.   - 1. Bone marrow reserve:   Absolute neutrophil count (ANC) ≥1.0 × 10³/μL (segmented and band neutrophils); Lymphocyte count >300/mm³; CD3+ T-cell count >150/mm³; Platelet count ≥50 × 10³/μL; Hemoglobin ≥100 g/L in the absence of blood transfusions within at least 2 weeks.   1. Reproductive potential:   Patients and their partners must use effective contraception throughout the study duration and for 12 months after cessation of the investigational treatment. They must either agree to employ two different highly effective barrier methods or practice total sexual abstinence throughout the study. |
| --- | --- |
| **Exclusion criteria** | 1. Previous treatment with any gene therapy product, including anti-CD19 cell therapy. 2. Central nervous system (CNS) involvement. 3. Patients who are candidates for and consent to autologous or allogeneic hematopoietic stem cell transplantation (HSCT). 4. Active hepatitis B or hepatitis C (HCV RNA-positive). 5. HIV-infected patients. 6. Uncontrolled acute bacterial, viral, or fungal infections that pose a life-threatening risk (e.g., positive blood culture within 72 hours prior to infusion). 7. Unstable angina and/or myocardial infarction within 6 months before screening. 8. Prior or concurrent malignancies, except for:   8.1.Basal cell or squamous cell carcinoma (adequate wound healing required before the study).  8.2.Carcinoma in situ of the cervix or breast, with no signs of recurrence for at least 3 years prior to the study.  8.3.Primary malignant tumor that has been completely resected and remains in full remission for ≥5 years.  9. Naïve T-lymphocyte count (CD3+CCR7+CD45RO-) ≤ 0,5%.  10. Pregnant and breastfeeding women.  11. Hypersensitivity to any of the excipients of the cellular product.  12. Cardiac arrhythmias not controlled by medication.  13. Patients with active neurological autoimmune or inflammatory disorders (e.g., Guillain-Barré syndrome, amyotrophic lateral sclerosis).  14. Presence of any primary immunodeficiency.  15. History of autoimmune diseases (except for thyroiditis).  16. Pollen or drug allergies (poli-allergy).  17. Socioeconomic or geographic circumstances that prevent proper adherence to the protocol requirements for treatment and subsequent observation (this should be incorporated into points 1 or 2). |
| **Withdrawal criteria** | 1. Individual intolerance to the study drug. 2. Patient’s wish to withdraw from the study. 3. Serious adverse events (SAEs) occurring during the study per CSAE criteria. 4. Violation of protocol conditions by the patient. 5. Detection of a second malignant tumor.   Additionally, patients who die from well-documented causes unrelated to the primary disease (e.g., infection, accident) or upon diagnosis of another malignant tumor are also considered withdrawn from the study. |
